# Supplementary material for: Deficit of mitogen-activated protein kinase phosphatase 1 (DUSP1) accelerates progressive hearing loss
Source: eLife. 2019 Apr 2;8:e39159. doi: 10.7554/eLife.39159 (PMC6464786; doi:10.7554/eLife.39159)
Supplement: Supplementary file 1. [file elife-39159-supp1.docx]

| **Supplementary File 1.** **Monthly ABR threshold (dB SPL)** | | | | | | | | | | | | | | | | | | | | | | | | |
| --- | --- | --- | --- | --- | --- | --- | --- | --- | --- | --- | --- | --- | --- | --- | --- | --- | --- | --- | --- | --- | --- | --- | --- | --- |
|  | **Age (month)** | | | | | | | | | | | | | | | | | | | | | | | |
|  | **1** | | | | | | | | **2** | | | | | | | | **4** | | | | | | | |
|  | *Dusp1^+/+^* | | | | *Dusp1^-/-^* | | | | *Dusp1^+/+^* | | | | *Dusp1^-/-^* | | | | *Dusp1^+/+^* | | | | *Dusp1^-/-^* | | | |
|  | Mean | ± | SEM | N | Mean | ± | SEM | N | Mean | ± | SEM | N | Mean | ± | SEM | N | Mean | ± | SEM | N | Mean | ± | SEM | N |
| **CLICK** | 18,3 | ± | 2,5 | 6 | 26,7 | ± | 1,1 | 6 | 19,8 | ± | 0,9 | 26 | 31,2 | ± | 1,5 | 25 | 18,6 | ± | 0,9 | 7 | 41,8 | ± | 3,1 | 14 |
| **28 kHz** | 21,7 | ± | 1,7 | 6 | 21,7 | ± | 1,7 | 6 | 23,3 | ± | 0,9 | 26 | 36,0 | ± | 2,5 | 25 | 25,0 | ± | 1,5 | 7 | 56,7 | ± | 3,3 | 15 |
| **40 kHz** | 23,3 | ± | 2,5 | 6 | 31,7 | ± | 2,1 | 6 | 28,1 | ± | 1,0 | 26 | 43,0 | ± | 1,5 | 25 | 32,1 | ± | 1,0 | 7 | 55,0 | ± | 2,9 | 15 |
|  |  |  |  |  |  |  |  |  |  |  |  |  |  |  |  |  |  |  |  |  |  |  |  |  |
|  | **5** | | | | | | | | **6** | | | | **7** | | | | **8** | | | | | | | |
|  | *Dusp1^+/+^* | | | | *Dusp1^-/-^* | | | | *Dusp1^-/-^* | | | | *Dusp1^+/+^* | | | | *Dusp1^+/+^* | | | | *Dusp1^-/-^* | | | |
|  | Mean | ± | SEM | N | Mean | ± | SEM | N | Mean | ± | SEM | N | Mean | ± | SEM | N | Mean | ± | SEM | N | Mean | ± | SEM | N |
| **CLICK** | 21,7 | ± | 1,2 | 15 | 25,0 | ± | 2,9 | 6 | 40,0 | ± | 2,0 | 4 | 21,1 | ± | 1,2 | 14 | 24,6 | ± | 2,2 | 12 | 53,8 | ± | 3,6 | 16 |
| **28 kHz** | 26,0 | ± | 1,2 | 10 | 26,3 | ± | 3,8 | 6 | 50,0 | ± | 11,4 | 4 | 24,6 | ± | 1,5 | 14 | 29,1 | ± | 2,7 | 11 | 70,6 | ± | 2,8 | 16 |
| **40 kHz** | 33,0 | ± | 1,1 | 10 | 37,5 | ± | 4,3 | 6 | 50,0 | ± | 2,0 | 4 | 33,2 | ± | 1,3 | 14 | 35,9 | ± | 3,5 | 11 | 63,4 | ± | 2,3 | 16 |
|  |  |  |  |  |  |  |  |  |  |  |  |  |  |  |  |  |  |  |  |  |  |  |  |  |
|  | **9** | | | | | | | | **12** | | | | | | | | **13** | | | |  |  |  |  |
|  | *Dusp1^+/+^* | | | | *Dusp1^-/-^* | | | | *Dusp1^+/+^* | | | | *Dusp1^-/-^* | | | | *Dusp1^-/-^* | | | |  |  |  |  |
|  | Mean | ± | SEM | N | Mean | ± | SEM | N | Mean | ± | SEM | N | Mean | ± | SEM | N | Mean | ± | SEM | N |  |  |  |  |
| **CLICK** | 30,0 | ± | 4,1 | 4 | 65,0 | ± | 2,2 | 6 | 47,5 | ± | 4,6 | 6 | 90,0 | ± | 0,0 | 2 | 90,0 |  |  | 1 |  |  |  |  |
| **28 kHz** | 36,7 | ± | 3,1 | 6 | 88,3 | ± | 1,7 | 6 | 60,0 | ± | 6,5 | 7 | 90,0 | ± | 0,0 | 2 | 90,0 |  |  | 1 |  |  |  |  |
| **40 kHz** | 45,0 | ± | 3,2 | 6 | 73,3 | ± | 3,3 | 6 | 58,6 | ± | 4,7 | 7 | 90,0 | ± | 0,0 | 2 | 100,0 |  |  | 1 |  |  |  |  |
