# Supplementary material for: Deficit of mitogen-activated protein kinase phosphatase 1 (DUSP1) accelerates progressive hearing loss
Source: eLife. 2019 Apr 2;8:e39159. doi: 10.7554/eLife.39159 (PMC6464786; doi:10.7554/eLife.39159)
Supplement: Supplementary file 2. [file elife-39159-supp2.docx]

| **Supplementary File 2. Taqman essays for RT-qPCR** | | |
| --- | --- | --- |
| **Symbol** | **Gene** | **Reference** |
| *Tgfβ1* | *Transforming growth factor, beta 1* | Mm01178820_m1 |
| *Il1β* | *Interleukin 1beta* | Mm00434228_m1 |
| *Il6* | *Interleukin 6* | Mm00446190_m1 |
| *Tnfa* | *Transforming nuclear factor* | Mm99999068_m1 |
| *Il10* | *Interleukin 10* | Mm00439614_m1 |
| *Foxp3* | *Forkhead box P3* | Mm00475162_m1 |
| *Gpx1* | *Glutathione peroxidase 1* | Mm00656767_g1 |
| *Gpx4* | *Glutathione peroxidase 4* | Mm00515041_m1 |
| *Gsr* | *Glutation reductasa* | Mm00439154_m1 |
| *Gss* | *Glutation sintetasa* | Mm00515065_m1 |
| *Gclc* | *Glutamate-cysteine ligase, catalytic subunit* | Mm00802655_m1 |
| *Gclm* | *Glutamate-cysteine ligase, modifier subunit* | Mm00514996_m1 |
| *Cbs* | *Cystathionine beta-synthase* | Mm00460654_m1 |
| *Nox3* | *NADPH oxidase 3* | Mm01339132_m1 |
| *Nox4* | *NADPH oxidase 4* | Mm00479246_m1 |
| *Ucp1* | *Uncoupling protein 1* | Mm01244861_m1 |
| *Cat* | *Catalase* | Mm00437992_m1 |
| *Cyba* | *Cytochrome b-245, P22phox* | Mm00514478_m1 |
| *Kim1* | *Kidney injury molecule 1* | Mm00506686_m1 |
| *Apaf1* | *Apoptotic peptidase activating factor 1* | Mm01223702_m1 |
| *Mpz* | *Myelin P0* | Mm00485141_g1 |
| *RbFox3* | *NeuN* | Mm01248771_m1 |
| *Sox2* | *SRY-box containing gene 2* | Mm03053810_s1 |
| *Slc26a5* | *Prestin* | Mm00446145_m1 |
