# Supplementary material for: Deficit of mitogen-activated protein kinase phosphatase 1 (DUSP1) accelerates progressive hearing loss
Source: eLife. 2019 Apr 2;8:e39159. doi: 10.7554/eLife.39159 (PMC6464786; doi:10.7554/eLife.39159)
Supplement: Supplementary file 3. — Primers for MKPs were designed using Primer Express 3.0 software and the mouse gene sequences available on the Ensembl genome database with references: NM_013642.3 (Dusp1), NM_010090.2 (Dusp2), NM_176933.4 (Dusp4), NM_001085390.1 (Dusp5), NM_026268.3 (Dusp6), NM_153459.4 (Dusp7), NM_008748.3 (Dusp8), NM_022019.6 (Dusp10) and NM_130447.3 (Dusp16). Base numbers indicate the location of the primer sequences in the corresponding mRNA; primers for Dusp1 were designed in the region of exon 2. [file elife-39159-supp3.docx]

| **Supplementary File 3. Primers for RT-qPCR** | | | | |
| --- | --- | --- | --- | --- |
| **Gene** | **Bases** | **Forward Primer (5'-3')** | **Bases** | **Reverse Primer (3'-5')** |
| *Dusp1 (Dusp1)* | 466-484 | AGTGCAGAATCCGGATGCA | 523-508 | CTGGGCCCCCCTGATC |
| *Dusp2 (Pac1)* | 395-412 | TGCGAGGCGGTTTCAAAA | 460-443 | CCTGGGCAGGAGCTTCAG |
| *Dusp4 (Mkp2)* | 761-779 | ACCACAAGGCCGACATCAG | 827-805 | CAGTCCTTTACTGCGTCGATGTA |
| *Dusp5 (Hvh3)* | 709-733 | GACATTAGCTCCCACTTTCAAGAAG | 783-764 | GACCAGGACCTTGCCTCCTT |
| *Dusp6* | 345-362 | CGGGCTGCTGCTCAAGAA | 409-389 | TGAAGCCACCTTCCAGGTAGA |
| *Dusp7* | 521-543 | TCCAAGGTGGTTTCAACAAGTTC | 588-567 | CGAGCTGTCCACGTTAGTCTCA |
| *Dusp8* | 218-237 | CTACACGGAGCCAGGTGGAT | 284-262 | TCTCGTGTGCTCTGGTCATACAC |
| *Dusp0* | 1126-1146 | GCCACAGACAGCAACAAACAG | 1200-1180 | CTGGTGAGCTTCCTCGATGAA |
| *Dusp16* | 505-522 | GGCTGCCAGCGAGATGTC | 569-546 | TTTAACACATAGCCAATCCCATTC |
